# p66Shc in Cardiovascular Pathology

**DOI:** 10.3390/cells11111855

**Published:** 2022-06-06

**Authors:** Landon Haslem, Jennifer M. Hays, Franklin A. Hays

**Affiliations:** 1 Biochemistry and Molecular Biology Department, College of Medicine, University of Oklahoma Health Sciences Center, Oklahoma City, OK 73104, USA; landon-haslem@ouhsc.edu (L.H.); jennifer-hays@ouhsc.edu (J.M.H.); 2 Stephenson Center, University of Oklahoma Health Sciences Center, Oklahoma City, OK 73104, USA; 3 Harold Hamm Diabetes Center, University of Oklahoma Health Sciences Center, Oklahoma City, OK 73104, USA

**Keywords:** mitochondrial dysfunction, p66Shc, apoptosis, ischemia/reperfusion, myocardial infarction, ROS, ShcA

## Abstract

p66Shc is a widely expressed protein that governs a variety of cardiovascular pathologies by generating, and exacerbating, pro-apoptotic ROS signals. Here, we review p66Shc’s connections to reactive oxygen species, expression, localization, and discuss p66Shc signaling and mitochondrial functions. Emphasis is placed on recent p66Shc mitochondrial function discoveries including structure/function relationships, ROS identity and regulation, mechanistic insights, and how p66Shc-cyt c interactions can influence p66Shc mitochondrial function. Based on recent findings, a new p66Shc mitochondrial function model is also put forth wherein p66Shc acts as a rheostat that can promote or antagonize apoptosis. A discussion of how the revised p66Shc model fits previous findings in p66Shc-mediated cardiovascular pathology follows.

## 1. Introduction

The p66Shc adaptor protein is a broadly expressed canonical scaffold protein involved in modulating intracellular signal response [1,2,3]. In addition to adaptor function, p66Shc is an oxidoreductase that produces reactive oxygen species (ROS) in a mitochondria-dependent manner [4,5,6,7]. p66Shc governs outcomes for many pathologies, including endothelial dysfunction, coronary artery disease (CAD), and ischemia/reperfusion injuries (IRI) [8,9,10]. During cardiovascular insults, p66Shc exacerbates ROS accumulation, which increases pro-apoptotic responses and worsens clinical outcomes. IRI clinical severity is closely tied to p66Shc expression and activity, such that p66Shc acts as a biomarker for CAD and IRIs [11,12,13]. Since ROS accumulation is the primary cause of p66Shc-mediated cardiovascular effects, its inhibition has high therapeutic potential in cardiovascular pathology. In this article we discuss, and review, the role that p66Shc plays in cardiovascular pathology. 

## 2. Reactive Oxygen Species, Aging, and Oxidative Stress

p66Shc mitochondrial function is coupled to ROS and ROS-mediated conditions such as aging and cardiovascular disease [14,15]. Free radical aging and oxidative stress theories posit that accumulated ROS-induced oxidative damage is central to aging and its associated pathologies [16,17]. Free radical aging and oxidative stress processes are characterized by consistent fitness loss as cellular processes become incrementally less efficient, leading to protein expression and functional alterations that cause cellular dysregulation [18]. However, this view of aging, and pathology etiology, was adapted because of the role ROS plays as a signaling molecule in routine cell functions [19]. Thus, excessive ROS or ROS dysregulation is key to free radical aging and oxidative stress.

In general, ROS are molecules that contain oxygen with higher reactivity profiles than O_2_ and are generated within cells by partial oxygen reduction [20]. ROS can exist as radicals (superoxide anion, hydroxyl radical) or as non-radical high-energy molecules capable of forming radicals (H_2_O_2_, organic peroxides). Radicals have unpaired/incomplete electron orbitals that increase their reactivity until stabilized by abstracting an electron from another molecule [21]. The distinction between radical and non-radical ROS is important as radicals are generally too reactive to travel outside of their production site, but non-radical ROS can [22,23,24]. Mobility differences between ROS forms can therefore affect and regulate subcellular redox signaling [25,26].

However, non-radical ROS can also generate radical ROS, which can affect intercompartmental signaling. A common example is known as Fenton chemistry, where H_2_O_2_ reacts with transition metal ions to produce hydroxyl radicals [27]. Excessive radicals cause damage by destabilizing or mismatching DNA, forming lipid peroxides or peroxyradicals, and oxidizing sulfur residues or carbonylating proteins [21,28,29,30]. These reactions can result in genomic, membrane, and protein instability that accumulate with age, which makes them aging biomarkers [31,32,33,34,35,36,37].

Within cells, there are several ROS sources. ROS are created by myeloperoxidase and NADPH oxidase (NOX) which form hypochlorous acid and superoxide anion, respectively. These proteins are associated with immune cells and have high activity in respiratory bursts that target pathogens [38,39]. However, non-immune cells have NOX homologues involved in ROS-mediated survival, growth, and apoptosis signaling [40]. Other ROS-generating proteins include: xanthine oxidoreductase (purine base breakdown), cytochrome p450 proteins (xenobiotic detoxification), and monoamine oxidase (dopamine breakdown) [41,42,43]. Peroxisomal fatty acid β-oxidation also produces H_2_O_2_, which enters the cytoplasm and may contribute to NF-kB and mTORC1 regulation [39,44]. Other common ROS generation sites include peroxisomes, lysosomes, endoplasmic reticulum, and the plasma membrane [45]. However, the primary cellular ROS source is mitochondrial respiration. This occurs when electrons leak between electron transport chain (ETC) complexes I and II or complexes II and III, reacting with oxygen to form superoxide anion [46,47,48,49]. Lastly, p66Shc is an oxidoreductase that directly contributes to pro-apoptotic mitochondrial ROS activity and indirectly to cytoplasmic ROS levels, which will be discussed in detail below [7]. p66hc-mediated ROS activity plays a central role in cardiovascular pathology through a variety of interacting pathways and is still emerging as a research field and potential therapeutic target in cardiovascular pathology, making it the focus of this review.

## 3. Reactive Oxygen Species—Neutralization, Necessity, and Pathology

Proper ROS function requires homeostatic balance. Excess ROS is damaging and promotes apoptosis, while deficiency prevents normal cell signaling required for growth and survival [50]. Although Excess ROS is pro-apoptotic, it is balanced by antioxidants which can be enzymes or small molecules. Known enzyme antioxidants can work independent of regenerating molecules but often require reducing equivalents from interacting proteins. Enzyme antioxidants that work independently include: catalase (reduces H_2_O_2_), glutathione S-transferases (reduce hydroperoxides), superoxide dismutases (reduce superoxide anion to H_2_O_2_), and peroxidase-active cytochrome c (oxidizes superoxide anion to O_2_). Enzyme antioxidants that work with interacting proteins include: glutaredoxins with glutathione (reduce disulfide bonds), thioredoxins with thioredoxin reductase (reduce disulfide bonds), peroxiredoxins with glutathione or thioredoxins (reduce peroxides), and glutathione peroxidases with glutathione/glutathione reductase (reduce H_2_O_2_ and peroxides) [51,52,53,54,55,56,57,58,59,60,61,62,63,64,65]. Most enzyme antioxidants have subcellular location-dependent isozymes with at least one form present in mitochondria [57,59,64,66,67,68,69].

Small molecules that contribute to redox homeostasis include vitamin C, vitamin E, and N-acetyl cysteine (NAC). Vitamin C is a water-soluble antioxidant that protects against cholesterol oxidation, scavenges dissolved O_2_, and neutralizes radical ROS, while vitamin E is a lipid soluble radical scavenger that can be regenerated by vitamin C [68,70,71,72]. NAC is a glutathione precursor with a redox active thiol that neutralizes ROS and can reverse deleterious disulfide formations [73].

Although ROS overproduction plays an important role in many pathologies, moderate ROS levels are required for optimal cell function. Technical limitations have prevented precise quantification of beneficial and detrimental ROS concentrations but it is widely accepted that ROS effects exist as a spectrum. Beneficial levels lie at an intermediate position in the spectrum but can fluctuate based on cell type and ROS activating stimuli, while excessive decreases and increases in ROS are harmful [20,24].

ROS play an important role in transducing environmental signals that dictate cell fate [19]. These redox signals are often transferred to downstream effectors via reversible protein modifications such as cysteine residue oxidation, affecting an array of proteins including phosphatases, kinases, transcription factors, and others [74]. Although, in theory, protein oxidation signaling reactions could be random, kinetics and solvent accessibility cause specific oxidation patterns in target proteins [50]. Since free radicals are restricted to their origination sites, H_2_O_2_ performs the majority of ROS signaling between cell compartments, often using peroxiporins to facilitate transfers [22,23,25,75,76,77,78,79]. However, it should be noted that ROS dysregulation (radical or non-radical) in a single compartment can affect whole-cell health, emphasizing the importance of subcellular compartmental ROS regulation [80,81]. 

ROS-activated pathways include nuclear factor-κB (NF-κB), phosphoinositide 3-kinase/AKT (PI3K/AKT), nuclear factor-erythroid factor 2-related factor 2/Kelch-like ECH-associated protein 1 (NRF2/KEAP1), and mitogen-activated protein kinase (MAPK) [25,82,83,84,85,86,87]. These pathways demonstrate the diverse effects of ROS signaling and provide insight into how ROS dysregulation can affect cells as these pathways regulate responses in inflammation, immunity, growth, survival, and apoptosis. In addition, downstream ROS effects differ by ROS concentration and stimulus source with low, moderate, and high ROS levels respectively favoring proliferation, differentiation, and cell death [19,88,89]. However, these are generalized trends and must be taken within the context of upstream signaling and local area or compartmental concentrations [80].

These pathways dictate many cell- and tissue-specific roles in pathology, making ROS-targeting therapies central to treatment strategies for many conditions. Some examples include neurodegenerative disease, aging, diabetes, organ failure, cancer, chronic obstructive pulmonary disease (COPD), endothelial dysfunction, sepsis, wound healing, and ischemia/reperfusion (I/R) injuries [8,14,18,31,90,91,92,93,94,95,96,97,98,99,100,101]. However, most treatments have not been directed towards location- or protein-specific ROS sources but rather whole-cell redox status and have been met with recurrent clinical failure. In fact, some treatments were harmful in cardiovascular pathologies [102]. Clinical failure of non-specific antioxidant administration is considered a result of neutralizing both physiological and pathological ROS [80,81,103,104,105]. Thus, emerging treatments to ROS-mediated pathology have shifted toward selective modulation of ROS sources dysregulated during pathology [16,81]. One of the proteins associated with dysregulated ROS in pathology is p66Shc, which was discovered as a longevity modifying protein that can promote apoptosis by generating ROS within mitochondria while also acting as a signal adapter at cell membranes.

## 4. p66Shc Discovery—Aging Research

**Figure 1:**
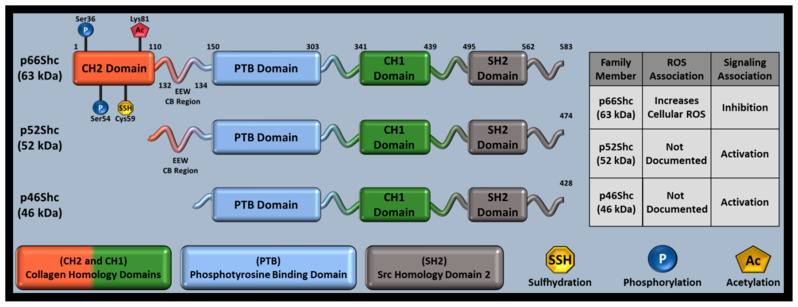
ShcA family modular structure and CH2 domain post-translational modifications affecting ROS output. All ShcA proteins have SH2, CH1, and PTB domains but differ by CH2 domain length. p66Shc has a unique set of promoters and is tied to oxidative stress or Ras inhibition. p52/46Shc share a set of promoters, do not have documented ROS activity, and promote cell cycle progression and differentiation. Important ROS-related PTMs include Ser36 phosphorylation (mitochondrial translocation), Ser54 phosphorylation (decreased proteasome degradation), Cys59 sulfhydration (decreased Ser36 phosphorylation), and Lys81 acetylation (increased Ser36 phosphorylation).

Interest in how Src homology 2 (SH2) domains mediate tyrosine receptor signaling led to the ShcA family’s discovery, which contains an SH2 domain in all family members (Figure 1) [106]. Tyrosine kinase receptors utilize adaptor proteins with SH2 domains at their cytoplasmic tails near receptor phosphorylation sites. SH2 domains can then recruit Grb2, another adaptor protein that promotes cell differentiation and growth via mitogen activated protein kinase (MAPK). ShcA transgene overexpression increased tumor incidence in mice, suggesting that ShcA proteins may activate MAPK. Although MAPK activation was later observed in response to p46Shc and p52Shc, p66Shc decreased mitogen signaling and was associated with increased ROS, separating p66Shc from p46Shc and p56Shc as a redox protein [107]. Further investigation identified that p46Shc and p52Shc share a transcript, while p66Shc has a unique transcript and N-terminal CH2 domain capable of replicating many p66Shc functions absent of its remaining domains [108]. 

When tested against UV and oxidative stress or increased tyrosine kinase signaling in mouse embryonic fibroblasts (MEFs), p66Shc was associated with cell death and altered electrophoretic movement, indicating that a post-translational modification (PTM), Ser36 phosphorylation, was associated with cell death [4]. Mouse p66Shc knockout (KO) studies corroborated p66Shc’s role as a redox protein that governs apoptosis and cell fate as p66Shc genetic removal increased mouse lifespan and tolerance to oxidative damage in stressed or pathological settings but had the opposite effect in unstressed settings [4,109,110,111,112]. These dichotomous effects may be explained by p66Shc ROS dysregulation since increased p66Shc-mediated ROS is consistent with worsened outcomes for many pathologies, but complete ShcA KO is embryonic lethal and moderate ROS is required for normal cell activity [19,113]. Promising results in lifespan and ROS attenuation have since led to many discoveries regarding p66Shc adaptor and oxidoreductase functions.

The original p66Shc KO mice experiments showed an around 30% increase in lifespan over WT mice and oxidative stress resistance, with no apparent negative effects [4]. Lifespan effects also appeared to be dose dependent as p66Shc^+/−^ mice live longer than WT but not as long as KO mice [4,111]. Upon further characterization, p66Shc KO mice also had decreased adipocyte triglyceride accumulation, increased metabolism, improved behavioral plasticity as adults, and have improved health at older ages [114,115]. These mice also show increased resistance to obesity, diabetes, ischemic insults, and atherosclerosis [115,116,117,118,119,120,121,122]. However, when p66Shc KO mice were moved to natural conditions, subjected to winter weather and food competition for one year, p66Shc KO mice had shortened lifespans [111]. This observation was consistent when low temperature and starvation conditions were mimicked within a controlled laboratory environment, killing 50% of the KO mice, and no WT mice. These results were accompanied with findings indicating adipose dysfunction [111]. Thus, p66Shc plays an important role in cardiovascular health, energy metabolism, and gerontology.

## 5. The ShcA Family 

The ShcA family consists of three members: p46Shc, p52Shc, and p66Shc. ShcA members are named by their approximate mass (~63 kDa, ~52 kDa, ~46 kDa) and domain composition, as well as Src Homology (SH) and Collagen homology (CH) domains. Despite their namesake only indicating SH and CH domains, all ShcA proteins also contain a phosphotyrosine binding (PTB) domain (Figure 1). P66Shc is the longest family member (583 residues), has its own set of promoters, and is primarily associated with oxidative stress and RAS inhibition while p52Shc (474 residues) and p46Shc (429 residues) share a set of promoters and are primarily associated with cell cycle progression and differentiation [3,108,123,124]. The family shares a single locus (1q21), producing different family members from 13 exons via alternative splicing and different start codons [125]. This results in ShcA proteins that share a single sequence, only differing by N-terminal amino acid length. The ShcA N-terminal domain is known as the CH2 (collagen homology 2) domain. P66Shc contains a full CH2 domain, p52Shc has a truncated CH2 domain, and p46Shc has no CH2 domain [125]. Functional differences between ShcA proteins are therefore CH2-mediated. All ShcA proteins function as signal adapters and undergo post-translational modifications (PTMs) that alter function, but p66Shc is the only protein member shown to produce reactive oxygen species (ROS), potentially explaining the divergent gene structure within the family and making p66Shc the most studied family member [7].

## 6. p66Shc Expression and Localization

p66Shc is expressed differentially throughout organs, but is expressed in adipocytes, lymphocytes, spleen, kidney, liver, lung, brain, and heart [114,115,126,127,128,129]. p66Shc’s differential expression led to p66Shc transcription and post-translation investigations that could explain expression patterns. Expressing p66Shc in cell lines not natively expressing p66Shc revealed that demethylating agents and deacetylases cause dose-dependent increases in p66Shc expression, suggesting that p66Shc’s promoter could be regulated via cysteine methylation or histone deacetylation [130]. Later studies revealed that low-density lipoprotein (LDL) could also increase p66Shc transcription via p66Shc promoter methylation at a CpG site [131]. p53, a transcriptional regulator and tumor repressor whose apoptotic function requires p66Shc, is associated with vascular disorders and increases p66Shc expression by binding p66Shc’s promoter region [132,133,134].

SIRT1 (histone deacetylase) also binds p66Shc’s promoter, decreasing p66Shc transcription via histone H3 Lys9 deacetylation. Similarly, SIRT1 overexpression in mice decreased p66Shc transcript and protein levels. However, SIRT1-mediated repression caused by SIRT1 overexpression can be overcome with high glucose and oxidized LDL in cell lines [135]. Further investigation indicated that prolonged high glucose causes a memory effect (sustained hypomethylation) on p66Shc’s promoter. This results in increased p66Shc and histone acetyl transferase (GCN5) expression, even after the original high glucose stimulus is normalized, while worsening diabetic endothelial dysfunction [136]. 

p66Shc’s expression is also affected by obesity-induced SUV39H1 (methyltransferase) downregulation. In these conditions acetyl transferase SRC-1 and demethylase JMJD2C are upregulated, resulting in decreased p66Shc promoter methylation and increased acetylation that increases p66Shc expression [137]. Of note, SIRT1 also protects SUV39H1 from ubiquitin-mediated degradation and may affect p66Shc expression through this interaction [138]. Thus, p66Shc expression is governed by transcriptional regulators and diet-inducible epigenetic factors.

In cancerous cells, nuclear erythroid 2-related factor 2 (Nrf2) can bind demethylated p66Shc promoter, increasing p66Shc transcription [4,139,140]. This finding is interesting because Nrf2 normally increases antioxidant responses and p66Shc’s traditional mitochondrial role is ROS production that leads to apoptosis; however, in this report, Ser36 phosphorylation did not increase mitochondrial trafficking as it has in other reported cell types [141]. These results suggest that p66Shc transcription and signaling may be cell dependent and that Ser36 phosphorylation may not be an exclusive signal for p66Shc mitochondrial translocation. 

PTMs also affect p66Shc expression. Oxidative stress can cause Rac1-mediated p66Shc phosphorylation at Ser54 and Thr386, increasing p66Shc stability by decreasing proteasome degradation [142]. Since adapter proteins function primarily in cytosol, p66Shc cellular localization may have been limited to cytoplasm; however, p66Shc was found unevenly distributed between mitochondria, cytoplasm, and endoplasmic reticulum [143,144]. The other ShcA members are found in the cytosol and either the endoplasmic reticulum (p52Shc) or mitochondrial matrix (p46Shc) [143,145]. In non-stressed cells that express p66Shc, p66Shc localizes throughout cellular compartments with the following subcellular pattern: endoplasmic reticulum (24%), cytoplasmic complex with Peroxiredoxin 1 (Prx1, 32%), and mitochondria (44%) [146,147]. However, a stressed cellular environment causes CH2 phosphorylation and increased mitochondrial trafficking (discussed in the following sections).

## 7. p66Shc Signaling Overview 

As an adaptor family, ShcA proteins are associated with signal transduction from varied stimuli (e.g., integrins, cytokines, and growth factors) and regulate downstream signaling for both physiological and pathological responses [148]. These observations support previous suggestions that p66Shc may provide a dual regulatory role as it can function as a proto-oncogene via growth factor signaling, or as an apoptosis regulator through pro-apoptotic mitochondrial ROS production (reviewed in detail in subsequent sections) [149]. Activated receptor tyrosine kinases recruit ShcA members with their cytoplasmic tails. When p52Shc or p46Shc bind receptor tails, they recruit the growth factor receptor-bound protein 2 and son of sevenless 1 (Grb2-Sos1) complex at p52Shc’s or p46Shc’s SH2 domain, activating Ras/MAPK mitogenic signaling [108,125]. However, p66Shc fails to induce mitogenic signaling and competes against the other ShcA members for Grb2, inhibiting potential MAPK signals [108,150,151]. p66Shc also inhibits extracellular-signal regulated kinase (ERK) signaling from insulin growth factor (IGF-1) and p52Shc is required for polyoma middle T antigen-induced hemangioma transformation in rats by promoting PI3K activation [143,152]. p66Shc Ser36 phosphorylation causes Grb2-Sos1 complex dissolution that leads to p66Shc/RAC1-mediated NADPH oxidase activation (via a Sos1/Eps8/E3b1 complex) and terminates Ras-MAPK and Ras-ERK signaling [108,153,154].

In addition, p66Shc increases the guanine exchange factor activity of Sos1 by competitively inhibiting Sos1-Grb2 interactions and increasing Sos1/Eps8/E3b1 complex formation to selectively promote Rac1 activity [155]. This action is primarily mediated by interactions between a PPLP motif in p66Shc’s CH2 domain and Grb2′s COOH-terminal src homology 3 domain [155]. In vitro assays did not directly assess other residues or PTM effects, but they may further promote Rac1 activation. p66Shc-mediated Rac1 activation is also associated with increased intracellular H_2_O_2_ production, likely via NADPH oxidase, and showed CH2 domain PPLP dependency [155]. These findings suggest that p66Shc may modulate many cellular functions regulated by Sos1 and Grb2, including cytosolic ROS production. Rac1 overexpression in PC-12 lines increased p66Shc expression, phosphorylation, and protein stability (half-life increased from 4.5 to 8 h) while decreasing ubiquitination [156]. These effects were reversed when p38MAPK was inhibited in PC-12 cells with SB203580 or SP600125, indicating that Rac1-mediated overexpression is governed by p38MAPK [156]. p38MAPK-dependent p66Shc overexpression was dependent on S54 (CH2 domain) and, to a lesser extent, T386 (CH1 domain) phosphorylation [156]. Intracellular Rac1-mediated ROS activity and oxidative stress-mediated apoptosis were also dependent on S54 and T386 hydroxyl group chemistry as cell lines coexpressing WT p66Shc and a p66Shc S54A/T386A double mutant generated less ROS than those overproducing p66Shc. Rac1 overexpression appeared to only affect p66Shc, not p52Shc or p46Shc [156]. However, Rac1 overexpression increased cellular ROS independent of p66Shc expression, suggesting that p66Shc serves as a non-essential adaptor protein that governs NADPH oxidase activity in these tests [156]. These functions are important in p66Shc cardiovascular pathology because overactive NADPH oxidase is associated with worsened IRI outcomes, decreased post-MI heart function, and heart failure [157].

Since phosphorylation patterns have ShcA signaling effects, phosphatase effects were also examined and showed that PTP-PEST interacts with p66Shc’s and p52Shc’s PTB domain, reducing MAPK activation under conditions that stimulate insulin signaling and antigen receptor stimulated lymphocyte activation [142,158]. Phosphatase and tensin homologue deleted at chromosome 10 (PTEN) is another phosphatase and tumor suppressor that dephosphorylates ShcA members and prevents ShcA-Grb2 binding, decreasing MAPK signaling and ShcA-mediated cell migration [159,160]. Protein phosphatase 2A (PP2A) and protein tyrosine phosphatase ε (PTPε) can also cause ShcA dephosphorylation and inhibit MAPK signaling [160]. Thus, p66Shc can inhibit mitogenic signaling or promote NADPH ROS-mediated apoptosis via its roles at the cell membrane. 

Yet, ROS also regulates Wnt signaling and the Wnt3a ligand causes p66Shc phosphorylation in endothelial cells and β-catenin dephosphorylation [161]. In these cells, p66Shc KD caused decreased β-catenin-dependent transcription but p66Shc overexpression decreased β-catenin dephosphorylation while increasing β-catenin-dependent transcription. Exogenous H_2_O_2_ also showed increased dephosphorylation and was inhibited by the non-specific antioxidant N-acetyl cysteine or catalase, collectively suggesting that p66Shc has an important role in ROS–Wnt signal integration [161].

Given the connection between oxidative stress and aging, p66Shc-mediated ROS production likely contributes to aging, but p66Shc’s ROS production is strongly associated with many other pathologies, particularly cardiovascular and ischemia/reperfusion pathology [162]. While the CH1 domain governs adaptor protein function, CH2 Ser36 (only present in p66Shc) phosphorylation is associated with p66Shc-mediated mitochondrial ROS generation [4]. Ser36 phosphorylation is primarily regulated by protein kinase β or protein kinase C (PKC-β, PKC), other proteins such as lectin-like oxidized LDL receptor 1 and PKC-β2, c-Jun N-terminal kinase (JNK) can also phosphorylate p66Shc’s Ser36 residue [153,163,164]. 

After p66Shc is phosphorylated at Ser36, the prolyl isomerase peptidyl-prolyl cis-trans isomerase NIMA-interacting 1 (PIN1) induces a p66Shc conformational change and, after a dephosphorylation event (potentially mediated by PP2a or PTPε), p66Shc enters the intermitochondrial space and can form complexes with the heat shock protein HSPA9 (mtHSP70, mortalin) [146,159,160]. The mortalin-p66Shc complex dissociates under high stress conditions, resulting in cyt c release, caspase 3 activation, and apoptosis [146].

Investigating p66Shc-mediated apoptosis revealed that p66Shc utilizes its unique CH2 domain to produce ROS, governed by possible dimer/tetramer formation that involves thiol-disulfide exchanges at Cys59 and cyt c interactions [5,124]. Tests with H_2_S supplementation or overexpression of proteins involved in H_2_S synthesis also showed that Cys59 S-sulfhydration can decrease p66Shc-PKCβII interactions, decreasing downstream ROS production, and indicated that sulfur-based therapeutics may affect p66Shc function via thiol-disulfide manipulations [165]. Surprisingly, the CH2 domain was able to induce ROS and cause mitochondrial swelling, independent of p66Shc’s other domains [124].

Since p66Shc increased mitochondrial ROS in an oxidative stress dependent manner, p66Shc’s definition expanded to include redox sensitive oxidoreductase apart from its adaptor protein role. Investigating SIRT1′s potential direct effects on p66Shc found that Lys81 acetylation (CH2 domain), which is increased in diabetic conditions or with SIRT1 knockdown (KD), is also required for p66Shc’s oxidoreductase function in hyperglycemic endothelium [166]. However, human umbilical vein endothelial cells (HUVECs) stimulated with vascular endothelial growth factor (VEGF) were still able to cause Ser36 phosphorylation, indicating that Lys81 acetylation effects are stimulus dependent. Preventing Lys81 acetylation via p66Shc Lys81Arg mutations inhibited Ser36 phosphorylation and mitochondrial ROS production, improving endothelial dysfunction and linking p66Shc PTMs to endothelial health. 

Apart from linking Ras to receptor stimuli and oxidoreductase function, the ShcA family also plays an important role in transducing mechanical stimuli. Complete ShcA KO is embryonic lethal (E11.5) and promotes severe cardiovascular tissue dysfunction with decreased contacts between neighboring cells and with extracellular matrix (ECM) [113]. This corroborates additional localization studies that identified p52Shc and p66Shc recruit focal adhesion kinase (FAK) at focal adhesion sites via their PTB domain [167,168]. Complete ShcA KO also causes decreased ability to spread on fibronectin, a sign of dysfunctional mechanotransduction [113].

Within endothelial cells, p52Shc interacts with a variety of integrins that increase ERK and Rac1 activity which promotes adhesion-dependent survival and all ShcA members respond to endothelial shear stress by interacting with α_v_β_3_ and β_1_ integrins [169,170]. Mouse models show that increased aortic arch shear stress leads to ShcA phosphorylation via EGFR2 signaling and interactions with the mechanosensory complex PECAM-1/VE-Cadherin/VEGFR2, leading to inflammatory responses [2]. Myocardial-specific ShcA KO in mice further explored ShcA’s role in mechanical coupling. Myocardial ShcA KO impairs systolic function while increasing myocardial contractility and causes ECM and collagen alterations, which was replicated by selectively inhibiting myocardial ShcA PTB domain [171]. Further evidence of ShcA’s involvement in mechanosensory functions was provided when investigators performed knock-in mouse studies that demonstrated that muscle spindles, which govern motor behavior, require phosphorylated ShcA CH1 domains to form [172].

p66Shc also contributes to ShcA mechanosensory function. p66Shc antisense blocking prevents the myoblast–myotube transition, a process that requires distinct cellular mechanical changes. The same researchers also observed decreased stress fiber structure and cell rounding in their study, indicative of severe internal tension loss [173]. In other studies, p66Shc KD showed decreased epithelial differentiation and increased proliferation, but decreased functions governed by mechanical stimuli, such as morphogenesis and mitosis arrest [174,175]. These findings suggested that p66Shc governs anoikis, which was later confirmed as a p66Shc-dependent process that is regulated by RhoA signaling [168,176]. On the contrary, anchorage-independent growth has been observed with RhoA suppression [177]. Tumor metastasis and cell line transformations resistant to anoikis are associated with decreased p66Shc expression, with ectopic p66Shc expression returning the cells to normal anoikis levels but only if focal adhesion targeting is restored [176]. Meanwhile, cells with high dependence on anoikis and mechanosensory information for normal function, such as endothelium and bronchial epithelium, also require p66Shc for sustained anoikis but lose anoikis function with p66Shc KD [176]. 

**Figure 2:**
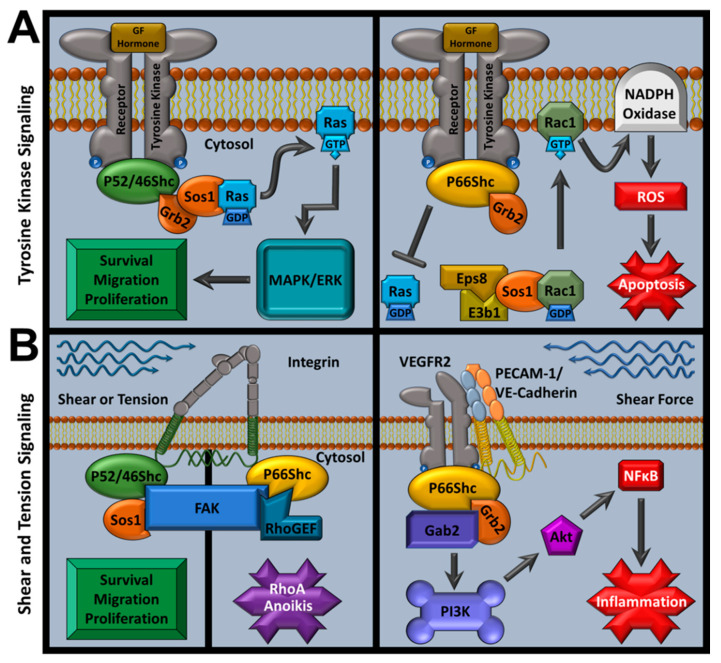
ShcA signaling summary. (**A**) ShcA responses to tyrosine kinase growth factor (GF) and hormone signals. P52/46Shc causes Ras-dependent MAPK/ERK signaling that promotes survival, migration, and proliferation; left panel. p66Shc inhibits Ras signaling by competitively increasing Rac1-mediated ROS generation via NADPH oxidase activation; right panel. (**B**) ShcA-mediated integrin and VEGFR2-Cadherin-PECAM responses to shear force or tension signals. Integrin shear or tension signals result in p52/46Shc pro-survival signaling via FAK-Sos1 while p66Shc leads to anoikis via RhoGEF-RhoA signaling; left panel. Endothelial VEGFR2-VE-Cadherin-PECAM-1 complexes respond to shear by increasing p66Shc-mediated inflammation via Gab2-PI3K signaling; right panel.

Both anoikis and cell proliferation after adhesion loss are mediated by the p66Shc/FAK/RhoGEF complex and are governed by matrix stiffness [167,178]. Similar to tyrosine kinase signaling, p52Shc and p66Shc have different effects in their mechanosensory roles. The p66Shc-FAK complex recruits Rho GEFs, while the p52Shc-FAK complex recruits Ras GEFs, altering Rho and Ras downstream signaling, respectively [179]. This provides context for how decreased p66Shc in test conditions or metastatic cancers can cause both increased cell survival and decreased anoikis. Despite p66Shc’s role in cell–cell adhesion and cytoskeletal signaling, which could influence cardiovascular pathologies such as angina perctoris or cardiac arrest, this aspect of p66Shc research is limited in the context of cardiovascular disease. However, it is plausible that p66Shc’s mechanosensory function contributes to atherosceloris, MI, and other pathologies as p66Shc KO improves their outcomes, but these outcomes are also associated with decreased p66Shc-mediated ROS activity and deconvoluting the interactions between p66Shc mechanical and ROS-activating stimuli is difficult [118,121,180,181,182]. p66Shc signaling pathways are summarized in Figure 2.

## 8. Classical p66Shc Mitochondrial ROS Activity Pathway

**Figure 3:**
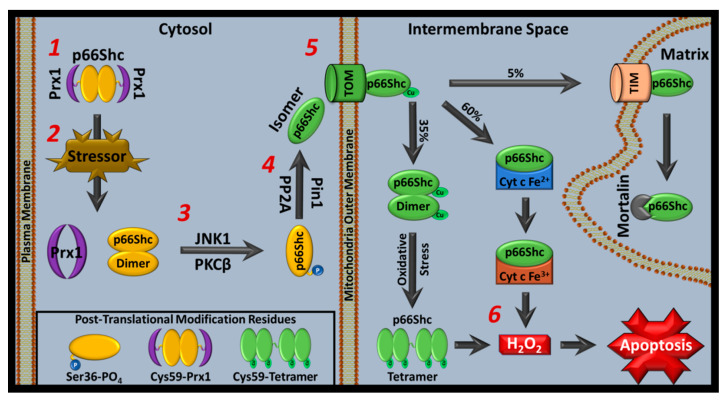
Classical p66Shc mitochondrial ROS activity pathway. (1) Until stressors are present, p66Shc remains inactive via Prx1-mediated cytosol sequestration. (2) Once stressed, Prx1 releases p66Shc as a dimer. (3) Free p66Shc dimers are phosphorylated by JNK1 or PKCβ, resulting in phosphorylated p66Shc monomers. (4) p66Shc monomers are isomerized and dephosphorylated via Pin1 and PP2a, respectively. (5) p66Shc isomers are granted IMS access via TOM interactions that cause copper to associate with p66Shc. (6) p66Shc oxidizes cyt c to produce H_2_O_2_. Oligomerization status is unclear during ROS generation, but purified mouse CH2 experiments and mouse embryonic fibroblast studies implicate monomers, dimers, and tetramers.

In response to cellular stress, p66Shc translocates into mitochondria. Once inside, p66Shc contributes to pro-apoptotic ROS accumulation causing caspase activation and apoptosis. However, several proteins influence p66Shc ROS activity by regulating p66Shc’s mitochondrial translocation [7]. In this section, we describe the classical pro-apoptotic p66Shc pathway and background on p66Shc ROS activity influencers (Figure 3).

### 8.1. Absent of Cell Stress, Peroxiredoxin 1 Prevents p66Shc Mitochondrial Translocation via Cytoplasmic Sequestration

Prx1’s role in p66Shc mitochondrial function is to sequester p66Shc within cytoplasm via direct binding interactions, mediated by p66Shc’s Cys59 residue. This prevents mitochondrial translocation until cell stress signals lead to p66Shc-Prx1 complex dissolution [147]. Peroxiredoxins are ubiquitous ROS regulators with location-dependent functions and are major contributors to metastasis progression. Peroxiredoxin 1 (Prx1) primarily resides in the cytoplasm and contributes to antioxidant processes via thiol-disulfide exchange where Prx1’s Cys52 and Cys173 become oxidized, producing an intermolecular disulfide bond and detoxified peroxides [183,184]. Reduced Prx1 can then be regenerated through various mechanisms [183,185]. In a common route for Prx1-mediated peroxide detoxification, thioredoxin (Trx) provides reducing equivalents to Prx1 that allows Prx1 to suppress ROS-mediated apoptosis driven by various proteins, including p66Shc [147,186,187,188]. However, oxidized Prx1 replaces peroxidase with other functions, including: chaperone, oncogene suppressing, immune enhancement, and ROS-dependent signaling [189,190,191,192,193,194,195,196,197,198,199,200].

In addition, Prx1 acts as a c-Abl tyrosine kinase inhibitor, an important cell death regulator that modulates p53 signaling and responds to oxidative stress, increasing JNK and MAPK pathway activation [192,194,201,202]. Prx1 also contributes to pro-apoptotic signaling by preventing PTEN inactivation caused by oxidation [183]. Furthermore, Prx1 associates with transcription factors important in cell death and growth such as NF-κB, androgen receptors, and p53 [203,204]. p53 is a key regulator of oxidative stress-induced apoptosis. Once activated, p53 increases pro-apoptotic protein expression, including p66Shc, causing caspase activation and mitochondrial apoptosis signals [205]. Prx1 mediates p53-regulated ROS-induced apoptosis, downstream of mammalian Ste20-like kinase-1 (MST1), which is phosphorylated during oxidative stress and causes increased proapoptotic signaling via MST1-forkhead box O3 (MST-FOXO3) interactions [206,207]. Thus, Prx1 contributes to the balance between pro-apoptotic and physiological ROS levels by direct ROS neutralization, inhibition of pro-apoptotic proteins that produce ROS (e.g., p66Shc), and transcriptional regulation over ROS pathways.

### 8.2. Cell Stress Causes Peroxiredoxin 1-p66Shc Dissociation, Allowing Protein Kinase c Family Members to Transduce Cell Stress Signals by Phosphorylating p66Shc at Ser36

The PKC family regulates p66Shc mitochondrial translocation and its activity is considered necessary for p66Shc to become ROS-active. In general, PKCs prime p66Shc for mitochondrial translocation via phosphorylation of p66Shc’s Ser36 in the CH2 domain. The PKC family has ten members that transduce many signals across a variety of pathways, including energy homeostasis and ROS production [208,209]. PKCs are categorized by their response to diacylglycerol (DAG) and other ligands downstream of GPCR-mediated phospholipase C activation. Their categories are conventional (requiring DAG, calcium, and phosphatidylserine), novel (requiring DAG and phosphatidylserine), and atypical PKCs (not affected by phosphatidyl serine or DAG) [208,210,211].

PKCβ is the PKC member most closely governing p66Shc’s ROS activity. PKCβ is a conventional PKC that is unique from other PKC family members as two isoforms are generated from a single locus via c-terminal exon alternative splicing, and each isoform (PKCβI and PKCβII) specializes in different roles [212]. PKCβI and PKCβII have similar primary structures but have differing C-terminal domains. PKCβ’s C-terminal domain is a catalytic center that is inactivated when its regulatory N-terminal domain binds the catalytic domain (resting state). When DAG binds PKCβ, these interactions are lost and PKCβ becomes active [213,214]. Once activated, PKCβ moves from cytosol to cell particulate fractions, which are mediated by lipid binding interactions that favor PKCβ’s active conformation [212,215,216,217].

Apart from DAG, PKCβ is also activated by PTMs including cysteine oxidation and tyrosine phosphorylation [218,219]. PKCβ has three constitutive phosphorylation sites. Their phosphorylation mechanisms are dependent on upstream kinase regulators, mTORC2, and PDK-1 [220,221]. After phosphorylation, PKCβ can interact with secondary messenger lipids but sustained activity results in PKCβ ubiquitination and degradation [222,223]. In addition, oxidative stress can activate PKCβ and may alter PKCβ cysteine chemistry, independent of calcium and DAG [224,225,226,227,228]. 

Since nutrient excess and obesity cause mitochondrial dysfunction, and because mitochondrial dysfunction causes ROS formation that activates PKCβ and increases p66Shc mitochondrial activity, obesity and p66Shc mitochondrial functions are linked [164,209,229,230,231,232,233,234]. PKCβ and p66Shc KO mice have increased metabolism, less insulin resistance, and heightened obesity resistance [114,115,120]. Along with causing p66Shc mitochondrial translocation, PKCβ activation inhibits autophagy and PKCβ KO or inhibition increases autophagy, a process that mitigates inflammation and must occur at low levels to maintain homeostasis [235,236,237,238,239]. Interestingly, exercise studies showed that exercise does not mitigate high fat diet-induced mitochondrial dysfunction, fat deposition, and insulin resistance in PKCβ KO mice while it does in WT mice [240]. Other reports suggest that PKCβ is downregulated with exercise, which decreases insulin resistance [241,242]. Since PKCβ and p66Shc function are coupled, it is not surprising that p66Shc also contributes to diabetic conditions [243].

Another PKC isoform, PKCδ, alters p66Shc mitochondrial activity. PKCδ is primarily located, in an inactive form, within cytoplasm, but a portion of PKCδ translocates to mitochondrial intermembrane spaces (IMS) where it can associate with p66Shc [143,244]. PKCδ is classified as a novel PKC isoform that binds p66Shc’s SH2 domain via a phosphorylated Tyr [245]. p66Shc-cyt c interactions activate PKCδ by facilitating site-specific PKCδ cysteine oxidation, independent of DAG [5,244]. Of note, this reaction causes p66Shc-mediated cyt c reduction and PKCδ oxidation. The reaction is catalyzed by vitamin A, which can bind PKCδ and cyt c in an orientation that facilitates this electron transfer [246,247,248,249,250]. However, PKCδ redox activation requires ferric cyt c, as respiration was only restored when ferric, but not ferrous, cyt c and retinol were added to isolated mitoplasts. Furthermore, respiration was not restored if p66Shc mutations preventing cyt c binding were present [246]. p66Shc KO MEFs also show lower glycolytic flux than controls, suggesting that p66Shc may alter metabolic pathways and contribute to ETC activity via cyt c reduction [122].

Further PKCδ experiments suggested a link between in vivo oxygen consumption and ATP synthesis upregulation via pyruvate dehydrogenase complex (PDHC) activation [246]. Although PKCε causes PDHC inhibition and localizes to mitochondrial matrices, it does not appear to directly interact with p66Shc [246,251]. p66Shc-mediated PKCδ redox-dependent activation may be reversible and would provide logical regulatory conditions for PKCδ activation via respiration activity [244]. High respiration increases ferrous cyt c pools while low respiration increases ferric cyt c pools, causing decreased or increased P66Shc-mediated PKCδ redox-dependent activation, respectively. Disrupting the p66Shc-vitamin A-PKCδ complex by decreasing p66Shc-cyt c or p66Shc-PKCδ interactions attenuates downstream signaling [245]. PKCδ also binds Raf near its DAG binding site and, as with its redox activation, requires vitamin A for Raf function [249,252,253,254]. Although other retinoids can bind in the same manner as vitamin A, vitamin A appears to the primary modulator of PKCδ function, with sustained activation from other retinoids causing cytotoxicity [249,251,255]. PKCδ KO and overexpression models are consistent with p66Shc KO models, where PKCδ overexpression caused insulin resistance and obesity while PKCδ KO mice were lean and had decreased insulin resistance [256,257]. This indicates that p66Shc ROS activity may govern PKCδ-related phenotypes.

### 8.3. Prolyl Isomerase 1 Interacts with p66Shc, Causing Conformational Changes That Prime p66Shc for Mitochondrial Translocation

After p66Shc’s Ser36 is phosphorylated, it can interact with prolyl isomerase 1 (Pin1), which induces phosphorylation-dependent cis–trans isomerization [164,258]. This step is a prerequisite to interactions with proteins that permit p66Shc IMS translocation. Pin1 is found in cytoplasm and nuclei but does not have a nuclear localization signal [259,260,261,262]. Pin1 expression correlates with increasing cell division but its regulation is not clear [259,261,263]. Pin1-induced transformational changes have strong biochemical effects on many proteins which may play an important role regulating cell growth and ROS-mediated diseases, such as Alzheimer’s and cancer [263]. Disrupting p66Shc–Pin1 interactions via Pin1 KO in MEFs decreases p66Shc translocation and shows increased resistance toward oxidative stress [164]. Similarly, Pin1 inhibition attenuates p66Shc ROS-mediated ischemia/reperfusion injuries in rat intestines [264].

Pin1 is also linked to mitosis regulation, with genetic constructs that decrease Pin1 function demonstrating mitotic arrest, cell cycle effects, or apoptosis [265,266,267,268,269,270,271]. These Pin1 depletion effects may be due to decreased phosphoprotein interactions, as Pin1 normally interacts with many pro-mitotic phosphoproteins, contributing to disorders like Alzheimer’s disease and cancer [263]. Protein phosphatase 2A (PP2A) specifically dephosphorylates the trans motif induced by Pin1 isomerization and has reciprocal effects to those observed by Pin1 in yeast studies [263,272,273].

### 8.4. p66Shc Is Dephosphorylated by Protein Phosphatase 2A to Allow Mitochondrial Translocation

In canonical p66Shc oxidoreductase mitochondrial function, Pin1-mediated isomerization is followed by dephosphorylation via PP2A interactions [7]. This set of reactions prepares p66Shc for mitochondrial entry by providing the required geometry for subsequent transport protein interactions. Although localization varies, in general, PP2A is dynamically localized between nuclei and cytoplasm. PP2A is a trimeric holoenzyme that can also function as dimer, with isoforms of each monomer determining localization and activity [274,275,276]. PP2A regulates oxidative stress, with increased PP2A activity improving oxidative stress-related outcomes in cardiovascular pathology, inflammation, and cancer [275]. PP2A is ubiquitously expressed and, in conjunction with PP1, represents ~90% of heart phosphatase activity [277,278]. It is also a therapeutic target for various diseases, including cardiovascular disease [279].

Decreased PP2A activity activates the ERK 1/2 signaling pathway (via increased ERK1/2, Akt, and glycogen synthase kinase 3β phosphorylation), an important predictor of IRI outcomes [280,281]. Yet, some IRI models have also demonstrated that these pathways are associated with increased PP2A activity [282,283,284]. One interpretation of these results is that PP2A activity fluctuates with time, as models collecting samples within 1 h and 6 h suggested that PP2A inhibition increases with time [285]. Potentially in agreement, PP2A therapeutic inhibition improved cardiac function when administered during reperfusion, but worsened outcomes if given during preconditioning [286].

Several reports suggest that PP2A activity is regulated by ROS, but they conflict in whether PP2A activity is upregulated or downregulated by ROS. An emerging view on these findings is that PP2A activity has tissue-dependent differential responses that vary in response to ROS concentration and time [275]. Data regarding PP2A during IRI is representative of this view as PP2A activity can be increased or decreased in IRIs, which may be caused by ROS fluctuations [275,287,288,289,290,291]. Furthermore, antioxidants and anti-inflammatory agents preserved PP2A expression in brain IRI models, improving outcomes and re-emphasizing ROS’s role in PP2A regulation [292]. In addition, PP2A inhibitors decrease infarct size more than preconditioning in rabbit ischemic heart models [293].

Since p66Shc produces ROS downstream of p66Shc-PP2A interactions, p66Shc mitochondrial translocation and ROS activity may have a causal link to PP2A activity, acting as a regulatory feedback loop that responds to cellular environments. PP2A activity and abundance decreases with increasing apoptosis, and excessive p66Shc mitochondrial activation causes ROS-mediated apoptosis.

### 8.5. p66Shc Enters the Intermembrane Space via Interactions with Mitochondrial Translocase of the Outer Membrane

The final step in p66Shc mitochondrial translocation is an interaction with the outer membrane translocase (TOM). p66Shc–TOM interactions place p66Shc in the IMS. Mitochondria have four distinct areas to which proteins can associate: mitochondrial matrix, inner mitochondrial membrane (IMM), intermembrane space (IMS), and outer mitochondrial membrane (OMM). Accurate protein targeting to each area can require specific machinery [294]. p66Shc utilizes TOM to reach p66Shc’s primary destination, the IMS, but a small portion of p66Shc interacts with the inner membrane translocase (TIM) to enter mitochondrial matrices where p66Shc forms complexes with mortalin that are destabilized by stress [146,295]. Although p66Shc reaches matrices, its matrix function(s) are not well-studied, and its ROS functions are tied to IMS localization.

TOM is a complex of seven subunits and two associated receptor subunits that form a channel in the OMM [296]. Of these subunits, p66Shc binds to Tom22 and Tom20 [295]. Tom22 and Tom20 play important roles in mitochondrial homeostasis, apoptosis, and mitochondrial health [297,298,299,300,301,302,303]. In general, proteins imported via TOM are recognized by Tom20, before interacting with Tom22, which permits IMS access [294]. Although IRI effects on TOM are not well-studied, researchers have shown that ischemia decreases Tom20 expression and that expression is rescued with ischemic preconditioning [304].

### 8.6. p66Shc Interacts with Cytochrome c, Causing H_2_O_2_ Accumulation, Permeability Transition Pore Formation, and Apoptosis

Once inside mitochondria, p66Shc traditional mitochondria function involves redox chemistry with cyt c, causing pro-apoptotic H_2_O_2_ accumulation. However, an alternative method for H_2_O_2_ accumulation mediated by p66Shc-cyt c interactions is discussed later in this review. Cyt c is a small, water soluble, heme c-containing protein that associates with the IMM outer leaflet and transports single electrons between complex III and complex IV of the ETC. This action promotes electron flux, ATP generation, and homeostasis [305]. In addition, cyt c can promote cell survival by decreasing mitochondrial ROS via superoxide anion scavenging or peroxidase activity, which is gained after interactions with cardiolipin (CL, an IMM lipid component comprising ~20–25% of the IMM) cause cyt c conformational changes that permit peroxide breakdown [51,52,53,54,55,56,306,307].

However, cyt c can also have pro-apoptotic functions. Under severe pro-apoptotic stress (e.g., ROS dysregulation) the IMM reorganizes, and endogenous reductant concentrations drop which causes cyt c to act as a CL-specific oxygenase [308,309,310,311]. This illustrates that CL-interactions govern cyt c’s dichotomous activity profile. CL oxidation is a critical step in pro-apoptotic signaling that produces various cell fate deciding saturated fatty acids, contributing to permeability transition pore (PTP) formation and cyt c release [311,312,313,314]. Released cyt c activates apoptotic protease activating factor – 1 (APAF-1) and associates with pro-caspase 9 to form the apoptosome, which activates caspase 3 and causes cell degradation [305,315,316].

According to one report, using overexpressed mouse CH2 domain, p66Shc exists primarily as a dimer that becomes pro-apoptotic after stress-induced Cys59-mediated dimer-tetramer transitions within mitochondria [124]. However, other studies suggest that a monomeric state is favored [146,295]. Regardless, stressed conditions lead to p66Shc pro-apoptotic H_2_O_2_ accumulation and permeability transition pore (PTP) formation, which is followed by cyt c outflow and caspase-mediated apoptosis [4,5,124,317]. Stressed conditions that increase p66Shc-derived ROS also increase p53 expression. In this state, PKCβ and JNK are activated to further increase p66Shc pro-apoptotic mitochondrial activity and glucose transport/actin polymerization is altered [5,318,319,320,321,322,323,324,325].

p66Shc also drives apoptosis by inhibiting antioxidant defenses, including superoxide dismutase (MnSOD, CuSOD, and ZnSOD), REF-1, and glutathione peroxidase via FOXO3a transcription factor downregulation [115,326,327,328,329,330]. Continued p53-mediated upregulation during high stress events may increase previously reported β_1_Pix/p66Shc/FOXO3a complex formation, sequestering FOXO3a to further promote apoptosis [331]. Although p66Shc-mediated apoptosis is associated with decreased antioxidant levels, decreases are not always observed [5,146,332,333]. Despite traditional p66Shc mitochondrial function being associated with H_2_O_2_ accumulation, some reports suggest that p66Shc produces superoxide anion, not H_2_O_2_ [90,334,335].

In total, previous studies suggest that p66Shc is a cell stress sensor that governs apoptosis, with Cys59 and Prx1 interactions inhibiting ROS activity in low-stress environments that are overcome by high stress which causes adverse effects in a variety of pathologies. Beyond potential Cys59 mediated interactions and cyt c effects, little was known about p66Shc’s ROS-generating mechanism or how parameters (e.g., pH) influence p66Shc ROS activity until recently (below).

## 9. Recent Advances in p66Shc Mitochondrial Function

### 9.1. p66Shc Oligomerization Status and Structure

p66Shc ROS activity models were previously built around the observation that overexpressed mouse-derived CH2 domains oligomerize [124]. It was suggested that a ROS-inactive cytoplasmic p66Shc dimer population was complexed with Prx1, which are monomerized and conformationally altered through several protein-mediated reactions before p66Shc monomers enter mitochondria. Once inside mitochondria, p66Shc was hypothesized to re-oligomerize via Cys59 interactions, and produce ROS, in a copper-dependent manner [7,124]. In this model, p66Shc could switch between dimer (reduced form) and tetramer (oxidized form), with normal conditions maintaining reduced dimeric p66Shc. Stress-associated antioxidant loss would then trigger tetramerization and subsequent ROS generation. However, these studies were performed with isolated CH2 domain, not full-length p66Shc (FLp66Shc). Furthermore, in vivo studies have not consistently reported p66Shc dimers or tetramers in their Western blot analyses, but monomers have been reported. 

Although p66Shc’s primary sequence and modular domain identities have long been known, little structural data has been collected on FLp66Shc despite its importance in understanding p66Shc activity. A recent study used analytical ultracentrifugation on a variety of human FLp66Shc and CH2 constructs, as opposed to mouse-derived CH2 domain. All constructs were monomeric and reducing agents did not alter oligomer status. However, sedimentation coefficient differences were observed between reducing and non-reducing conditions, indicating disulfide bond presence. Mass spectroscopy experiments identified that although Cys59 does not mediate intermolecular disulfide bonding, it plays a central role within an interdomain thiol-disulfide exchange network that governs p66Shc ROS production (below). Interestingly, CH2 (amino acids 1-110) was aggregation-prone and polydisperse, but including distal cyt c binding domain residues reversed this trend. These results suggest that FLp66Shc conformational dynamics could be regulated by distal CH2 amino acids. Molecular dynamics simulations and in silico structural models supported these findings, indicating that p66Shc has high conformational variability and is intrinsically disordered with interspersed regions of organization [182].

### 9.2. p66Shc ROS Identity and Regulation

Despite the traditional model that p66Shc produces H_2_O_2_, studies have also associated p66Shc with increased superoxide anion levels [90,334,335]. As discussed above, radical and non-radical ROS have different reactivity and signaling profiles, with radical ROS primarily functioning at its origination site and non-radical ROS responsible for inter-organelle signaling [21,22,23]. ROS composition differences can therefore govern compartmental and inter-compartmental redox signaling, altering cell fate [25,26]. A variety of functional assays have shown that p66Shc does not directly produce H_2_O_2_, but rather superoxide anion [182]. Further investigation determined that neither metal cations, nor cyt c, were needed for superoxide anion activity, even though both were previously considered requirements [5,90]. Purified CH2 domain did not bind copper of either valency. However, an alternative route for p66Shc-cyt c mediated H_2_O_2_ accumulation was identified that resolves discrepancies in observed ROS forms generated by p66Shc mitochondrial activity (below).

ROS therapy is undergoing paradigmatic shifts from increasing cellular reductant concentrations that change whole-cell redox state to targeted approaches focusing on compartmental ROS dysregulation [80,81,103,104]. Since p66Shc is dysregulated with increased ROS activity during several pathologies, understanding how p66Shc ROS activity is regulated could improve outcomes for p66Shc ROS-mediated pathology. Several environmental factors regulate p66Shc superoxide anion production during in vitro assays, such as: pH, temperature, shear, and cardiolipin presence. p66Shc ROS production is highest in acidic, warm, cardiolipin-rich environments but is also increased with direct shear (indirect shear on cell membranes also increases p66Shc mitochondrial translocation) [2,182]. Of note, CL is essential for normal electron transport and promotes respiratory complex assembly and could therefore affect structural characteristics of other proteins [336,337,338].

Conditions increasing superoxide anion activity were associated with increased α-helical secondary structure and the CH2 domain shows a larger increase in structure than FLp66Shc under high-activity conditions. Since conditions with optimal p66Shc pro-apoptotic ROS activity are commonly found in pathology (e.g., IRI), these observations suggested that p66Shc acts as a stress biosensor whose activity is upregulated by pathological environmental effects. Furthermore, p66Shc’s mitochondrial-specific ROS activity may be a result of non-IMS conditions having a relative inhibitory effect.

The first p66shc-selective ROS inhibitors have also been identified: idebenone and carvedilol. Carvedilol is a non-selective adrenergic blocker that is prescribed for heart failure and is a World Health Organization essential medicine that improves post-myocardial infarction (post-MI) remodeling; however, how carvedilol improves remodeling is not well-understood [80,339,340,341,342,343]. Carvedilol binds and inhibits p66Shc at nM concentrations (K_d_ ~ 48 nM, IC_50_ ~ 118 nM), which agrees with previous carvedilol-mediated lipid peroxidation inhibition values [344,345]. Of note, carvedilol is thought to provide added benefit over other adrenergic blockers because of its ability to inhibit ROS [344,346,347]. With the understanding that non-specific ROS inhibition fails to improve or worsens cardiovascular outcomes and that targeted ROS inhibition can improve outcomes, carvedilol-mediated p66Shc ROS inhibition may play a role in observed cardiovascular clinical outcomes [80,81,103,104]. On the other hand, idebenone has antioxidant properties and acts as a coenzyme Q mimetic. It is prescribed in Europe for mitochondrial diseases with p66Shc associations [348,349]. At high concentrations (40–80 µM), idebenone sensitizes PTP opening and inhibits ETC complex I, but p66Shc-idebenone affinity has a reported Kd of ~229 nM (IC_50_ ~ 328 nM) and idebenone’s low concentration mitochondrial mechanism of action has not previously been characterized [182]. Idebenone has also been shown to improve insulin sensitivity via p52Shc signaling activity [350]. These compounds were tested in a fish p66Shc KD cryoinjury MI model, and show promising p66Shc-mediated improvements in many parameters [182].

p66Shc superoxide anion activity is also modified by protein binding partners. Mortalin forms complexes with p66Shc, which is released as a monomer in a stress-dependent manner and is upregulated with oxidative stress. Therefore, mortalin may prevent p66Shc-mediated apoptosis via p66Shc binding and inhibition [146,351,352,353]. Mortalin-p66Shc binding affinity (K_d_ ~ 858 nM) and ROS inhibition was recently confirmed, corroborating previous findings where p66Shc release from its mortalin complex increases PTP formation, ROS, and cyt c release [146]. These findings also support reports where mortalin overexpression reduces ROS and provides IRI protection [354]. Although originally viewed as a mitochondrial-specific protein, mortalin localization studies demonstrate that mortalin is found throughout cells, including within cytoplasm [355]. In addition, mortalin inhibits p53 transcription, decreasing p66Shc expression via cytoplasmic p53 sequestration [132,133,134,356,357]. Since Prx1 governs p66Shc via cytoplasmic sequestration, and mortalin regulates p66Shc activity via p53 sequestration (decreasing p66Shc transcription), it follows that mortalin could have a second regulatory level over p66Shc involving cytoplasmic sequestration, such that mortalin works with Prx1 to prevent dysregulated p66Shc mitochondrial translocation [147].

### 9.3. p66Shc Superoxide Anion Mechanism

Although p66Shc’s superoxide anion production mechanism is not fully characterized, recent insights into p66Shc function have been published. Cys59-mediated thiol-disulfide interactions were previously implicated in p66Shc’s oxidoreductase activity as a potential ROS inhibiting interaction (p66Shc-Prx1 translocation inhibitory complex), but were also associated with intermolecular disulfide bond formation between purified mouse CH2 domains [124,147]. However, human FLp66Shc mass spectrometry studies indicate that Cys59 is the center of an intramolecular interdomain thiol-disulfide exchange network [182]. Both reports indicate that Cys59 is critical to conformational alterations that affect ROS production.

In FLp66Shc, Cys59 (CH2 domain) interacts with either Cys287 or Cys292 (PTB domain). Cys287 can also interact with Cys196 (PTB domain) while Cys292 also interacts with Cys574 (SH2 domain). Mutating any single Cys residue was less effective at inhibiting ROS production than a combination of C287/C292S mutation. This double mutant prevents Cys59 from interacting with either of its partners and shows precipitous p66Shc ROS inhibition. These findings are consistent with earlier Cys59 investigations that identified Cys59 as a sulfhydration site that decreases p66Shc-derived ROS upon sulfhydration. Cys59 sulfhydration was observed with H_2_S administration or when overexpressing proteins contributing to H_2_S synthesis [165]. The previously reported Cys59S/E132Q/E133Q mutant with decreased pro-apoptotic activity also showed stark ROS inhibition in these FLp66Shc constructs [5,182]. Since shear increases thiol-disulfide interchange, shear may increase superoxide anion production via enhanced intermolecular thiol recycling [358,359]. Furthermore, increased p66Shc superoxide anion production in acidic conditions suggests that thiol-disulfide interchanges may be a rate limiting step in ROS generation and regulated by electrophilic strength of leaving groups during interchange [360].

Cys59S/E132Q/E133Q mutations did not appear to prevent ROS activity based on lost thiol–disulfide interaction partners alone as molecular dynamics simulations using the triple mutant found that the EEW motif in the CH2 domain, or cyt c binding domain, form salt bridges and cation-π interactions with Arg177, Arg285, and Lys279. These interactions cause partial folding that alters the PTB’s N-terminal orientation, which also affects CH2 orientation. Since these salt bridges could be affected by environmental effects that alter p66Shc activity, they may regulate environmental effects on superoxide anion production [182]. Consistent with this notion, ShcA constructs showed increased alpha helical secondary structure when exposed to CL, which increased p66Shc superoxide anion production. CL also protected p66Shc from thermal denaturation and increased post-thermal melt secondary structure recovery, relative to controls.

The same study also identified a crucial amino acid for p66Shc-mediated superoxide anion generation, Tyr10. When CH2 constructs were reversibly acetylated at Tyr10 or if FLp66Shc constructs were point mutated to FLp66Shc Tyr10Ala, superoxide anion production was abrogated. Acetyl group removal restored ROS activity. Thus, Tyr10 may participate in an electron relay, which is further supported by molecular dynamics simulations that indicate close proximity between the CH2 domain and a putative electron sink in the PTB that is governed by interdomain interactions [361]. Of note, hypoxic conditions terminated ROS production, which may provide insight into why hypoxic conditions are beneficial in pathology associated with p66Shc ROS overproduction, such as MI and Leigh syndrome [362,363,364].

As Tyr radicals are important in many redox enzymatic reactions (e.g., cyt c oxidase) and because ROS-active ShcA constructs also displayed a 410 nm absorption peak (associated with Tyr radicals), p66Shc superoxide generation may be radical-mediated [365,366,367]. This hypothesis was supported by in vitro fluorescence assays conducted under conditions that increase radical formation, which showed increased p66Shc superoxide anion production. 

### 9.4. p66Shc-Cytochrome c Interactions

p66Shc-cyt c reaction descriptions were originally limited and suggested that p66Shc produced H_2_O_2_ via copper-dependent cyt c oxidation, mediated by Cys59 oligomerization [4,5,124]. However, reported p66Shc and cyt c reduction potentials do not support spontaneous p66Shc-mediated cyt c oxidation and there are no known pathways energetically coupled to support this reaction [5,368]. In addition, p66Shc has been shown to reduce cyt c as part of the PKCδ/retinol signalosome. In PKCδ/retinol signalosomes, PKCδ is activated by p66Shc-mediated cyt c reduction that causes PKCδ oxidation, which is catalyzed by vitamin A. Testing oxidized or reduced cyt c addition, combined with vitamin A, showed that respiration was restored with oxidized but not reduced cyt c in a signalosome-dependent manner [244]. Similar tests, performed with only p66Shc and cyt c, demonstrated that p66Shc reduces, not oxidizes, cyt c. In these in vitro experiments, human FLp66Shc or CH2 is capable of reducing but not oxidizing cyt c, independent of other proteins [182]. Since oxidized cyt c is required for normal cytoplasm-mitochondria transport via the mitochondrial disulfide relay function, p66Shc’s cyt c reductase function may also play a role in regulating mitochondrial protein import [369,370,371].

Several findings suggest that ShcA cyt c reductase function is independent of superoxide anion activity. p66Shc-mediated cyt c reduction was not inhibited by SOD’s presence nor by hypoxia, which abrogates superoxide anion fluorescent signals in superoxide anion production assays. p46Shc, which does not have reported ROS activity, also showed some reductase activity, though it was lower than p66Shc. In addition, when idebenone or carvedilol (p66Shc ROS inhibitors) are added to p66Shc-cyt c redox assays, cyt c reduction is increased [182].

Corroborating the above report where mitoplast respiration was dependent on PKCδ/retinol signalosome-mediated cyt c reduction, recent experiments demonstrated that p66Shc alone can increase complex IV and complex V activity when added to mitoplasts [182,244]. WT (ROS-active), Tyr10Ala (ROS-inactive), and Cys59Ser FLp66Shc mutants all increased Complex IV and Complex V ETC activity, corroborating a recent study that showed increased ETC activity with p66Shc activation in CNS cells [97]. Thus, p66Shc can increase ETC activity, independent of p66Shc’s superoxide anion activity. Although data suggest that Complex IV/V enhancement occurs through cyt c recycling, it is not clear whether enhancement occurs by direct p66Shc-mediated ETC redox chemistry or if electrons are passed from reduced cyt c to the ETC. 

Yet, p66Shc-cyt c interactions have a well-documented H_2_O_2_ accumulation effect, which was thought to occur via cyt c oxidation [5,372]. If p66Shc reduces cyt c, there must be an alternative mechanism for p66Shc-mediated H_2_O_2_ accumulation. An alternative mechanism was described where human FLp66Shc is able to non-competitively inhibit WT cyt c peroxidase function (K_I_ ~ 1.43 µM), resulting in decreased H_2_O_2_ degradation. Constitutively peroxidase active His26Tyr mutant cyt c was also inhibited by FLp66Shc (K_I_ ~ 3.54 µM) and shows inhibition values similar to minocycline-mediated cyt c His26Tyr peroxidase inhibition (K_I_ ~ 1.0 µM) [373]. In addition, purified human CH2 domain also caused a decrease in H_2_O_2_ breakdown (~48.9% signal decrease). Yet, p66Shc-cyt c binding experiments demonstrate that FLp66Shc has a ~7.7-fold higher binding affinity for WT cyt c than its peroxidase active His26Tyr mutant, suggesting that non-stressed conditions may favor an interaction that prevents cyt c peroxidase transformation and pro-apoptotic H_2_O_2_ accumulation [182].

p66Shc was also found to have another function that may influence apoptosis, caspase inhibition. p66Shc-mediated ROS overproduction leads to PTP formation and mitochondrial cyt c release, which ultimately causes caspase cascade activation and cellular destruction [5,151,372]. p66Shc-mediated caspase inhibition was tested in three cell lines: HS27, PCS-201, and WI-38. P66Shc significantly inhibited caspase 3/7 activity (~3-fold) and caspase 9 activity (~5-fold) but did not inhibit purified caspase 3 activity [182]. However, since p66Shc upregulation is associated with apoptosis these results suggest that caspase inhibition may be a cytoprotective effect against transient stress spikes and caspase leakage rather than a function that occurs during apoptosis.

In summary, p66Shc directly contributes to mitochondrial ROS accumulation via superoxide anion production, superoxide anion spontaneous dismutation to H_2_O_2_, and cyt c peroxidase inhibition. It should be noted that pro-apoptotic p66Shc ROS production is associated with stressed or pathological conditions, but not physiological conditions. P66Shc also performs mitochondrial functions associated with cell survival: ETC enhancement, caspase inhibition, and cyt c reduction. Thus, p66Shc appears to act as a biosensor promoting cell survival until stress thresholds are met, leading to pro-apoptotic signaling. 

### 9.5. Revised p66Shc Mitochondrial Function Model

**Figure 4:**
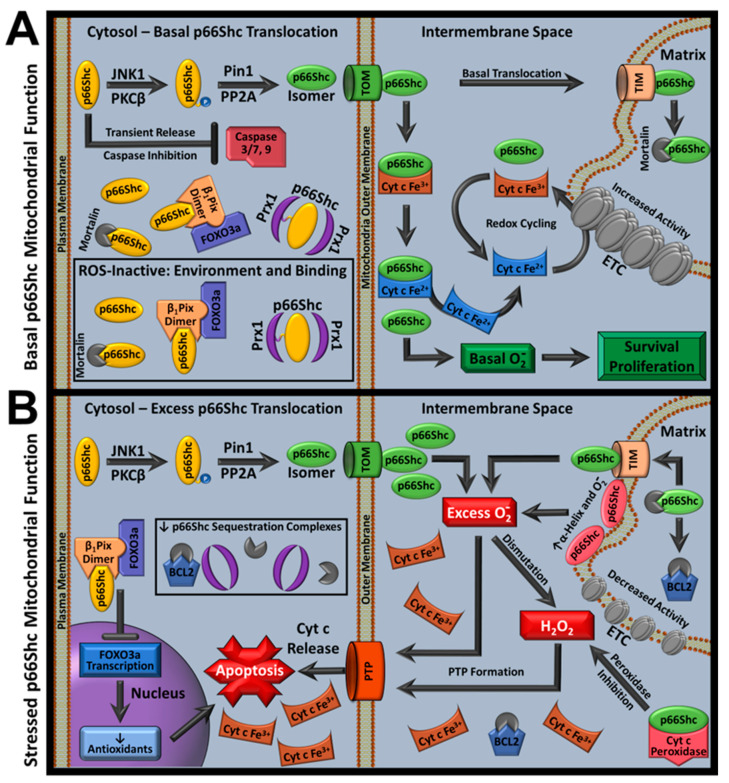
Revised p66Shc mitochondrial function model. (**A**) Basal p66Shc mitochondrial function. Within cytoplasm, p66Shc is ROS-inactivated by environmental effects and complexes that sequester p66Shc by preventing Ser36 phosphorylation (mortalin-p66Shc, β_1_Pix-FOXO3a-p66Shc, and Prx1-p66Shc complexes). Free cytoplasmic p66Shc can also inhibit caspase 3/7 and caspase 9 activity, promoting survival against transient caspase leaks from short-term stress. Meanwhile, p66Shc reduces cyt c and enhances ETC activity, potentially via redox cycling. In addition, p66Shc is ROS-activated by the IMS environment, producing basal superoxide anion levels that are required for normal cell survival and proliferation functions. (**B**) p66Shc in stressed cytoplasm and mitochondrial matrix is released from sequestration complexes and enters the IMS at increased amounts through p66Shc’s classical translocation pathway (as illustrated in Figure 3), as a monomer, until p66Shc reaches the IMS. Increased p66Shc translocation causes excess superoxide anion production, which is increased when p66Shc undergoes conformational changes (increased α-helix) by interacting with cardiolipin. Accumulating ROS leads to increased cyt c peroxidase levels, which p66Shc inhibits, causing further H_2_O_2_ accumulation that is exacerbated by superoxide anion spontaneous dismutation. Once a threshold is reached, PTP formation occurs, leading to cyt c release and apoptosis. Overall cell antioxidant pools are also exhausted during this process as p66Shc prevents FOXO3a antioxidant transcription by retaining FOXO3a outside of nuclei.

Given the above insights into p66Shc mitochondrial function, a new stress-dependent p66Shc model has been proposed that unifies previous and current findings (Figure 4). In the new model, during low-stress or physiological conditions, p66Shc favored functions are correlated with cell survival. Low-stress cytoplasmic p66Shc pools are ROS-inactivated by environmental factors and mortalin or Prx1 complexes. Meanwhile, mitochondrial p66Shc pools produce physiologic superoxide anion signals, prevent cyt c-mediated lipid peroxidation by stabilizing peroxidase-inactive cyt c conformations, and promote Complex IV/V activity in the ETC. 

However, high-stress conditions favor p66Shc’s pro-apoptotic functions. High-stress releases p66Shc from Prx1 and mortalin complexes, leading to increased mitochondrial translocation. Translocation activates p66Shc superoxide anion production by introducing favorable environmental conditions for ROS production. Increased ROS-active mitochondrial p66Shc concentrations cause increased mitochondrial ROS accumulation. This contributes to antioxidant depletion, cyt c peroxidase formation, and CL oxidation. If the stress stimulus is not corrected, heightened CL oxidation causes cyt c IMM disassociation and CL depletion that disrupts ETC function while p53 mediates p66Shc upregulation. p66Shc upregulation propagates pro-apoptotic signals by inhibiting FOXO3a-mediated antioxidant transcription. In these conditions, p66Shc strictly promotes apoptosis, as ongoing processes associated with stress stimuli, such as acidosis in IRI, further enhance p66Shc ROS activity and decrease availability to pro-survival pathways as PTP formation begins (e.g., depleted IMM cyt c availability and cyt c–ETC interactions), leading to cellular degradation [182]. 

p66Shc has both pro-apoptotic and pro-survival functions linked to mitochondrial ROS activity. P66Shc pro-apoptotic functions are apparent in high-stress or pathological models but not in unstressed models. This matches general cellular ROS trends, where high stress is correlated with ROS dysregulation and pathology but moderate ROS levels are required for healthy cells. The coordination between cell stress, ROS, and p66Shc are better exemplified by the revised p66Shc mitochondrial function model than the previous model. This model also unites and resolves paradoxical p66Shc findings such as those demonstrating that decreased p66Shc expression or ROS production improves IRIs and increases mouse lifespan in non-stressed environments, but p66Shc KOt models decrease ischemic preconditioning benefits, increase short-term myocardial injuries, and decrease mouse lifespan in natural environments [4,13,90,109,110,111,374,375].

In addition, this model is supported by a set of experiments testing whether the balance between p66Shc’s pro-survival related functions and pro-apoptotic functions could be tipped to increase pro-survival functions in zebrafish cryoinjury MI models. The experiments utilized p66Shc WT fish that were given p66Shc selective ROS inhibitors and compared them to p66Shc KD fish. p66Shc KD and ROS inhibitor groups showed improvements in many parameters with a concomitant decrease in myocardial ROS production. Some improvements include increases in post-MI body mass and physical activity while demonstrating decreases in inflammatory cell recruitment, fibrotic tissue area, platelet aggregation, and injury area. Furthermore, proteome analysis was consistent with caspase cascade inhibition and energy metabolism alterations contributing to improved MI outcomes. Lastly, non-selective ROS inhibition via NAC administration proved detrimental, corroborating the proposed benefits of targeted ROS inhibition [80,81,103,104,182]. In addition, the revised p66Shc model is also under clinical investigation as a cancer treatment (NCT04928508). However, in this instance, p66Shc’s balance is pushed toward pro-apoptotic functions by disrupting the p66Shc–mortalin complex via SHetA2 administration [376,377,378].

## 10. p66Shc’s Mitochondrial ROS Activity Role in Cardiovascular Pathology 

As noted above, p66Shc ROS production is negatively associated with many pathological states, indicating that inhibiting p66Shc ROS activity may improve their outcomes [118,119,379,380]. Some examples include neurodegenerative disease, aging, diabetes, organ failure, cancer, chronic obstructive pulmonary disease (COPD), endothelial dysfunction, sepsis, wound healing, and ischemia/reperfusion (I/R) injuries [31,90,91,92,93,96,97,98,100,101]. In this section, we review p66Shc-ROS mediated effects on cardiovascular pathology. Although p66Shc also plays an important role in metabolism, cytoskeleton/mechanosensory, and inflammatory functions, a detailed discussion of p66Shc’s role in these functions in cardiovascular pathology is beyond the scope of this review [113,114,115,120,169,170,174,175,176,177,178,179,182,350]. However, previous sections do include key interactions between p66Shc and these aspects of cardiovascular disease. 

### 10.1. p66Shc in Ischemia/Reperfusion Injuries

Heart disease is a substantial global healthcare and financial burden. In the U.S. alone, heart disease is diagnosed in 10.6% of adults, attributed to 23.1% of deaths, costs USD 351.2 billion annually (2014–2015), and reports suggest that heart disease burdens are rising [381,382]. The most common heart disease is coronary artery disease (CAD) which narrows arterial diameter, decreasing blood flow beyond plaques. If poorly managed, CAD transitions into myocardial infarction (MI). MI pathology is characterized by dysfunctional mitochondria that reverse their ATP generating activity with ATP expenditure and that pump protons into the IMS under ischemic conditions [383]. Mitochondrial dysfunction can be reversed if myocardium is reperfused within ~20 min, but extended ischemic episodes cause permanent damage that is exacerbated by reperfusion and linked to ROS dysregulation, PTP formation, and apoptosis [162,383,384,385,386,387,388].

p66Shc’s role in ischemia reperfusion injuries (IRIs) varies depending on ischemia and reperfusion duration but has been traditionally viewed as pro-apoptotic independent of IRI location, and has almost exclusively been studied in p66Shc genetic KO models as no p66Shc-selective therapeutics had been described until recently. However, some studies analyze p66Shc in PKC-β inhibition or KO models. p66Shc deletion improved IRIs in hind limb ischemia models, showing improvements in tissue viability and capillary density via decreased p66Shc-mediated ROS accumulation [118]. Other hind limb ischemia models revealed that p66Shc KO improved wound healing in a ROS-dependent manner, indicating that p66Shc ROS balance may regulate ischemic wound healing [389].

In mouse cardiomyocytes, p66Shc KO increased resistance to angiotensin II-mediated apoptosis and showed decreases in IRI-induced oxidative stress which were not further decreased by antioxidant administration, indicating p66Shc ROS dysregulation in ischemic cardiac tissue [119,121]. Cardiac IRI models showed that p66Shc mitochondrial translocation did not increase with short-term ischemia alone or with short-term ischemia and reperfusion, but did increase with long-duration ischemia followed by reperfusion [380]. In addition, p66Shc ROS inhibition via pharmaceutical Complex I or PKC-β inhibition decreased p66Shc Ser36 phosphorylation and improved outcomes [380]. However, others found that short-term ischemia was worsened by p66Shc deletion, underscoring p66Shc’s dual role in cardiac IRI [110]. p66Shc’s protective role in short-duration ischemia, which could be viewed as a transient cell stress spike, is further supported by studies reporting reduced ERK/Akt-mediated growth factor signaling in p66Shc KO fibroblast lines that decreased normal fibroblast activity [390]. Furthermore, p66Shc IRI cytoprotective effects were also observed when p66Shc KO decreased survivor activating factor enhancement and reperfusion injury salvage kinase pathways, with concomitant increases in mitochondrial swelling and caspase-3 mediated apoptosis in mouse cardiac IRIs, indicating that p66Shc had a pro-survival role in these conditions [110].

Interestingly, mouse cardiac specific p66Shc KO studies revealed that p66Shc KO does not notably affect heart rate, heart mass, or blood pressure, but cardiomyocyte counts were increased despite having a similar ventricular wall size [121]. This may reflect p66Shc’s neonatal role in heart development, since neonatal p66Shc KO cardiac tissue has increased SOD activity and decreased ROS-mediated apoptosis that could manifest as increased cells in the adult heart [391].

p66Shc IRI has also been studied in stroke models and shows similar trends to cardiac IRIs. For example, p66Shc KD after ischemic insult in mice preserved vessel integrity at the blood–brain barrier and increased survival rates while decreasing ROS levels, neurological loss, and lesions [13]. The same study showed that acute ischemic stroke patients had increased p66Shc expression in peripheral blood monocytes that correlated with neurological outcomes. Yet, p66Shc is required for ischemic preconditioning neuroprotective effects [392].

p66Shc’s role in post-MI effects is less studied than initial infarct effects. However, p66Shc KO has shown improved post-MI healing with decreases in fibrosis measurements and cardiac rupture [374]. The same report demonstrated that these effects were mediated by increased collagen formation and decreased matrix metallopeptidase 2 (MMP2) activation. MMP2 expression has also been shown to increase or decrease with p66Shc overexpression or deletion, respectively [393]. Since MMP2 activates TGFβ, and because its activity also correlates with p66Shc expression, p66Shc may regulate TGFβ activation [394]. This potential connection is strengthened by TGFβ activation mechanisms including ROS exposure, ionizing radiation, shear, and acidic pH, which also increase p66Shc superoxide anion production and pro-apoptotic signaling [182,395,396]. Thus, improved post-MI remodeling effects from p66Shc KO may be secondary to decreased TGFβ activation, which is strongly activated in MI and regulates post-MI remodeling [397].

Due to its high morbidity and mortality, pharmaceutical treatments that can mitigate initial MI and post-infarct remodeling have been frequently researched. Yet, few drugs with pre-clinical effectiveness are beneficial in MI patients and none were previously described as a p66Shc ROS inhibitor [398,399]. Of the promising therapeutics, most are related to p66Shc’s functions. For example, Coenzyme Q (CoQ) and mitoquinone (mitoQ), a CoQ variant with increased mitochondrial localization, both target the respiratory chain and attenuate ATP loss during I/R while scavenging excess ROS. These treatments are associated with improvements in: post-MI contractility, cardiac function, oxidative damage, cell death, and I/R mitigation during cardiac grafts, potentially via decreased lipid peroxidation. CoQ and mitoQ treatment also decrease pro-apoptotic cyt c release [400,401,402,403,404,405]. In fact, mitoQ completed clinical trials that showed cardiovascular benefits and is undergoing clinical trials for a variety of ROS-related conditions, including heart failure and kidney disease. A functionally similar drug, mito-TEMPO, scavenges mitochondrial superoxide and shows improvements in heart failure and diabetic cardiomyopathy models by increasing contractility and decreasing heart dilation, cell death, and oxidative stress [406,407,408,409]. 

Bendavia (also known as MTP-131 or SS-31), is a tetra-peptide that binds CL, preventing ROS-induced CL oxidation and cyt c peroxidase formation [410,411]. Bendavia decreases oxidative stress, infarct expansion, apoptosis, and detrimental heart remodeling while increasing ETC Complex I/IV (other ETC complexes were not tested) activity and decreasing infarct sizes in sheep and guinea pig models [412,413,414]. Lastly, resveratrol treatment activates SIRT1/SIRT3 and reduces post I/R infarct size [415]. Since SIRT1 activation decreases p66Shc expression, these results suggest that decreased p66Shc expression is beneficial in IRI [90,135,416]. 

Thus, promising IRI therapeutics are tied to increasing p66Shc pro-survival signaling or decreasing p66Shc pro-apoptotic ROS activity. As noted above, the first-described p66Shc selective ROS inhibitors have already been tested in zebrafish MI models and show improvements in a variety of clinically translatable parameters [182]. Although consistency in mammals remains to be seen, these represent a novel approach to p66Shc ROS-mediated pathology, including MI treatment, that decreases initial infarct damage in MI models and may improve recurrent MI as fibrosis measurements were substantially decreased in treated fish.

In addition, targeted superoxide anion inhibition could improve post-MI heart failure because post-MI heart failure is accompanied by a significant increase in free radical-mediated lipid peroxidation and plasma superoxide anion levels, but decreased catalase, glutathione, and SOD plasma levels [417,418]. These trends are further enhanced with heart failure severity, which also correlate with decreased antioxidant pools and heart function in heart failure patients [417,419,420].

### 10.2. p66Shc in Endothelial Dysfunction

Age-induced endothelial dysfunction is characterized by decreased NO production and decreased ability to increase lumen diameter, which corresponds with oxidative stress; however, aged p66Shc KO mice do not exhibit these impairments [107,333,421,422,423,424,425]. p66Shc KO mice in this study also had decreased age-related superoxide anion and nitrotyrosine (formed when NO and superoxide anion react) levels. p66Shc also inhibits NO generation as p66Shc KD led to increased markers of endothelial NO synthase activity and aged p66Shc KO mice showed similar results [426,427]. Thus, p66Shc governs endothelial ROS and NO production, which can affect many other pathologies.

Diabetes increases p66Shc expression (see expression and localization section). This links p66Shc to many diabetes-related conditions, including diabetes-induced endothelial dysfunction. p66Shc KO mice exhibit protection against diabetic endothelial dysfunction, oxidative stress, cardiomyopathy, and glomerulopathy [116,332,428]. Although p66Shc did not protect against diabetes induction or hemoglobin glycosylation, KO mice had lower nitrotyrosine and lipid peroxidation levels than their WT counterparts. Diabetes also causes advanced glycation end product accumulation, which promotes inflammation and ROS production via p66Shc ser36 phosphorylation. p66Shc KO mice also have decreased renal damage, circulating oxidative stress markers, and oxidative stress associated with glycation products [429,430,431]. In addition, glycation products increased p66Shc Ser36 phosphorylation in HEK-293 cells, depleting antioxidants and suppressing FOXO transcription. Furthermore, hyperglycemia increases p66Shc expression and decreases signaling from IGF1-stimulated phosphoinositide-3 kinase, AKT, which decreased cell survival in smooth muscle cells [432]. Thus, hyperglycemic p66Shc upregulation may upregulate p66Shc beyond its beneficial basal activity such that decreasing p66Shc is beneficial at rest for some cells.

Hyperlipidemia promotes coronary artery disease (CAD), a condition that promotes endothelial dysfunction, and increases oxidative damage in vasculature [433,434,435,436,437,438,439]. p66Shc KO mice on an apoliprotein E KO background showed that p66Shc also contributes to atherosclerosis, a common MI risk factor and CAD precursor [180,181]. Dyslipidemia can further exacerbate p66Shc ROS-mediated endothelial dysfunction and other pathologies as LDL increases p66Shc transcription via p66Shc promoter hypomethylation in endothelial cells [131]. 

Interestingly, CAD severity correlates with peripheral blood monocyte p66Shc mRNA concentrations and oxidative stress in humans, and p66Shc expression is therefore a CAD biomarker [11,12]. CAD patients also have thicker carotids that are less able to dilate in response to blood flow and the severity of dilatation resistance increases with increasing p66Shc mRNA [440]. Although p66Shc KO did not alter lipid concentrations in mice fed a high fat diet, KO mice showed improvements in lesion area, with decreases in systemic ROS, tissue ROS, and apoptosis [120]. Of note, all ShcA proteins are phosphorylated by shear stress, which is perpetually higher in CAD, which leads to inflammatory signaling [2,441]. Thus, p66Shc KO models are also causing increased p52/46Shc shear signaling that promotes survival and growth that is no longer balanced by pro-apoptotic p66Shc shear signaling (Figure 2). Therefore, observed improvements in p66Shc KO CAD models cannot be viewed exclusively as results from decreased p66Shc mitochondrial ROS production. 

## 11. Future Perspectives and Summary 

Although p66Shc’s full mechanism for superoxide anion production remains unanswered, recent advances have provided critical insight that can be utilized to further investigate p66Shc mechanistic details. An important mechanistic detail requiring further investigation is identification of upstream p66Shc electron donors in cyt c reduction. Since p66Shc binds the ubiquinone mimetic, idebenone, ubiquinone is a potential electron donor. However, many potential electron donors with reduction potentials favoring cyt c reduction exist within cells and, given p66Shc’s binding promiscuity, this reaction may occur through a variety of donors.

Furthermore, there are questions concerning how p66Shc modulates Complex IV/V activity of the ETC. Although the data infer that ETC activity is enhanced via cyt c recycling, direct electron donation to Complex IV has not been negated. Detailed structure–function experiments are also missing that could explain the step-wise process for superoxide anion production and whether the cysteine network directly contributes to electron flow or just conformational dynamics that permit superoxide anion production. Despite correlations between metabolism and p66Shc inhibition, targeted metabolism studies and mitochondrial oxygen consumption studies are also required to determine their mechanistic impacts on pathology. Lastly, p66Shc-CL effects are similar to those seen with cyt c and CL because both promote ROS production, but this correlation may have broader impacts that could be revealed with detailed analysis. 

Once elucidated, these details could provide the information required to inhibit mitochondrial pro-apoptotic p66Shc functions while keeping pro-survival functions intact. Current data suggest that specifically preventing cyt c peroxidase inhibition and superoxide anion production could provide substantial benefit to p66Shc-mediated cardiovascular disease. As indicated in previous sections, p66Shc-selective ROS inhibitors have been described and improve outcomes in zebrafish cryoinjury-induced MI. However, concomitant inhibition of p66Shc-mediated superoxide production and H_2_O_2_ accumulation, if administered at the proper time and concentration to avoid potential side effects, could provide greater cardioprotection than observed with p66Shc-selective ROS inhibition alone. On the contrary, increasing these functions can also provide benefits to many cancers. In addition, specifically increasing pro-survival functions in cardiovascular pathology may have similar benefits to p66Shc-selective ROS inhibition. The opposite approach can be taken with pro-survival functions as well, as decreasing p66Shc pro-survival signals could have a substantial impact on p66Shc-mediated cancers. 

The potential health benefits of modulating both sides of p66Shc’s cell fate functions (pro-survival and pro-apoptotic) are substantial but many obstacles have prevented such innovation. For example, until carvedilol and idebenone were described as selective p66Shc inhibitors, the primary method of studying p66Shc effects was genetic ablation, (despite being a drug target since 1999), which removes both p66Shc signaling and mitochondrial functions and makes data interpretation difficult. The newly discovered p66Shc-selecitve ROS inhibitors also have potential convoluting effects. Although binding and inhibition constants indicate that these newly described inhibitors are selective for p66Shc, they may have other targets influencing their results. For example, carvedilol also acts as a non-selective adrenergic blocker that may overlap with p66Shc inhibition. Idebenone is less likely to have overlap with other functions (e.g., Complex I inhibition) as they require concentrations ~170-340-fold higher than required for p66Shc binding and inhibition, respectively. However, second-generation inhibitors could be designed around idebenone and carvedilol to provide greater specificity for p66Shc. A complete p66Shc crystal structure is critical to produce second-generation p66Shc ROS inhibitors, as well as first-generation p66Shc pro-survival activators, but p66Shc’s inherent conformational variation and intrinsically disordered regions present a significant hurdle to crystallization. Furthermore, complete characterization of p66Shc’s superoxide and ETC-enhancing mechanisms will require intensive physical biochemistry experiments whose design, execution, and analysis will be difficult. Lastly, p66Shc largely behaves like a membrane protein during purification and overexpression, increasing the difficulty of performing high-throughput methods. 

Defining p66Shc activity is a complex process, given its broad range of functions, localization dynamics, location/environment effects on activity, and multiple isoforms. Yet, this complexity provides p66Shc with the flexibility required to act as a molecular rheostat of apoptosis. The refined p66Shc molecular rheostat model resolves previous discrepancies between research groups by uniting findings in a singular model that is supported by in vivo MI models and is under active clinical investigation for cancer studies. The revised model also provides new therapeutic targets with the potential to modulate a variety of pathological conditions by adjusting p66Shc’s rheostatic setpoint.
